# Characteristics of patients with myofascial pain syndrome of the low back

**DOI:** 10.1038/s41598-024-61319-5

**Published:** 2024-05-24

**Authors:** Pao-Feng Tsai, Joseph L. Edison, Chih-Hsuan Wang, Michael W. Gramlich, Kailea Q. Manning, Gopikrishna Deshpande, Adil Bashir, JoEllen Sefton

**Affiliations:** 1 College of Nursing, Auburn University, 710 S Donahue Dr, Auburn, AL 36849 USA; 2 Edward Via College of Osteopathic Medicine, Auburn, AL USA; 3 College of Education, Auburn University, Auburn, AL USA; 4 College of Science and Mathematics, Auburn University, Auburn, AL USA; 5 Samuel Ginn College of Engineering, Auburn University, Auburn, AL USA; 6 School of Kinesiology, Auburn University, Auburn, AL USA; 7 Auburn University Neuroimaging Center, Department of Electrical and Computer Engineering, Auburn University, Auburn, AL United States; 8 Department of Psychological Sciences, Auburn University, Auburn, AL United States; 9 Alabama Advanced Imaging Consortium, Birmingham, AL United States; 10 Center for Neuroscience, Auburn University, Auburn, AL United States; 11 Department of Psychiatry, National Institute of Mental Health and Neurosciences, Bangalore, India; 12 Department of Heritage Science and Technology, Indian Institute of Technology, Hyderabad, India

**Keywords:** Chronic low back pain, Myofascial pain syndrome, Trigger point, Diseases, Signs and symptoms

## Abstract

The objective of this study is to determine characteristics of patients with myofascial pain syndrome (MPS) of the low back and the degree to which the low back pain in the patients examined can be attributed to MPS. Twenty-five subjects with myofascial trigger point(s) [MTrP(s)] on the low back participated in this cross-sectional study. The location, number, and type of selected MTrPs were identified by palpation and verified by ultrasound. Pain pressure threshold, physical function, and other self-reported outcomes were measured. Significant differences were found in Group 1 (Active), 2 (Latent), 3 (Atypical, no twitching but with spontaneous pain), and 4 (Atypical, no twitching and no spontaneous pain) of participants in the number of MTrPs, current pain, and worst pain in the past 24 h (*p* = .001–.01). There were interaction effects between spontaneous pain and twitching response on reports of physical function, current pain, and worst pain (*p* = .002–.04). Participants in Group 3 reported lower levels of physical function, and higher levels of current pain and worst pain compared to those in Group 4. Participants in Group 1 and 2 had similar levels of physical function, current pain, and worst pain. The number of MTrPs is most closely associated with the level of pain. Spontaneous pain report seems to be a decisive factor associated with poor physical function; however, twitching response is not.

## Introduction

Myofascial pain syndrome (MPS) is a regional pain disorder that causes small nodules of tight tissue called myofascial trigger points (MTrPs). Definitions of active and latent MTrPs varies^1,2^ but patients with active MTrPs typically present with spontaneous and recognizable pain. Latent MTrPs may produce local or referred pain after palpation and demonstrate myofascial dysfunction^3^. It has been hypothesized that mechanical factors, such as poor posture, abnormal gait, and prior injury result in muscle overload and eventually lead to muscle contraction and formation of MTrPs and pain reaction^2,4^. Alternative theories postulated that psychological factors, such as stress, are major elements contributing to the activation and maintenance of MTrPs and play a significant role in the intensity of the perception of pain^2^. This stems from the observation that myofascial pain is referred pain and hence, central sensitization may have a role to play in that process^5^.

Diagnosis of MPS currently depends on physical examination and patient’s self-report of pain. Lack of clinician training and skill, inconsistent diagnosis criteria and terminology, and subjectivity of diagnosing may result in ignoring patient symptoms and misdiagnosis^6–8^. In addition, patients may not feel that it is necessary or important to report symptoms due to the chronic nature of the pain. Therefore, the prevalence rate of MPS is unclear. It is estimated that 30% of patients visiting primary care clinics and 85% visiting pain clinics suffer from MPS^3,9,10^. Approximately 30–93% of patients with widespread pain also present with MTrPs^3,10^. Specifically, studies found that 63.5–90% of patients with low back pain suffered from MPS^11,12^. Like most chronic pain conditions, MPS patients are likely undertreated^13^.

A few small studies have investigated the characteristics of patients with MPS of the low back as well as characteristics of MTrPs on the low back^11,14^. Patients’ demographics seem similar to patients with non-specific low back pain^11^. A study comparing low back pain and healthy adults found that individual patients showed multiple MTrPs on the low back and the intensity of their low back pain episode was associated with the number of active MTrPs measured^14^. This study neither confirmed the presence of trigger points using an ultrasound procedure nor compared the characteristics of patients with active and latent trigger points.

These past studies showed that there is a lack of clear understanding of MPS of the low back and the association between characteristics of MTrPs, such as spontaneous pain and twitching, and manifestation of MPS (such as poor quality of life, catastrophizing, self-efficacy, Kinesiophobia, depression and poor exercise motivation) in patients with current or past low back pain history. MPS may be the cause of lower back pain. Alternatively, patients with lower back pain can evolve into MPS or the low back pain may resolve. Therefore, the objective of this study is to determine characteristics of patients with myofascial pain syndrome (MPS) of the low back and the degree to which the low back pain in the patients examined can be attributed to MPS.

## Materials and methods

### Sample

This study was part of a larger cross-sectional study that investigated manifestations of MPS at the individual level (N = 25) and the characteristics of MTrP in the muscle region. The characteristics of MTrPs in the muscle region was published elsewhere^15^. The current study only reports the individual level data. This project was approved by Auburn University’s and Edward Via College of Osteopathic Medicine’s institutional review board. Additionally, all methods were performed in accordance with the relevant guidelines and regulations. Data was collected between 8-30-2021 and 6-30-2022. Inclusion criteria included participants who (1) self-reported having chronic low back pain either currently or in the past with palpable MTrP(s) on the low back muscles and hypo-echogenicity of MTrP region(s) confirmed by ultrasound examination, (2) were English-speaking, and (3) were ambulatory without a cane or walker. Exclusion criteria included having (1) major illness, such as cancer, (2) major surgery within 6 months, (3) major psychiatric disorder, such as bipolar disorder and depression, (4) cognitive impairment, or (5) other painful conditions of the lower back. Participants were recruited from the local community using a snowball sampling procedure. Informed consent was obtained from all participants.

*Identifying location, number, and type of MTrPs.* To screen for MPS, the participant was asked if he/she had low back pain in the past, had pain that comes and goes or had experienced pain at the time of the study. If the answer was yes, he/she was further examined to identify the site and number of MTrPs. Participants were positioned prone on an examination table. To identify the site and number of MTrPs, the participant was examined by an osteopathic doctor using physical examination and palpation to determine the presence or absence of MTrP(s). A total of 25 participants were screened. Three MTrPs were selected among the types of MTrPs for each participant for further verification by ultrasound. Priority is given to active MTrPs, followed by latent MTrPs, atypical MTrPs with spontaneous pain but no twitching, and atypical MTrPs without spontaneous pain and also without twitching. An ultrasound procedure using the Sonosit Edge II system was applied to confirm the palpation findings of the three MTrPs for each participant^15^. Findings showed 100% of them with hypo-echogenicity of MTrP region(s).

Based on the physical examination and palpation, we categorized MTrPs by the following criteria^15^: Category (1): a tender spot within a taut band of a skeletal muscle on the low back, a local twitch response, and spontaneous local or referred pain, discomfort, soreness, restricted movement, or other related symptoms identified. We defined this category as active MTrP. Category (2): Same as Group 1 except for spontaneous local or referred pain, discomfort, soreness, or other related complaints. We defined this category as latent MTrP. Category (3): Same as Group 1 except for twitch response. We defined this category as atypical MTrP. Category (4): A taut band within a skeletal muscle on the low back identified, but no other syndromes. We also defined this category as atypical MTrP.

Based on the types of MTrPs, participants were divided into four groups for analysis:

1. Group 1 (active MTrP group): participants had at least one active MTrP.
2. Group 2 (latent MTrP group): participants had no active MTrPs but had at least one latent MTrP.
3. Group 3 (atypical MTrP group): participants had no active and no latent MTrP but at least one nodule on the low back muscle was visible in the ultrasound exam and had spontaneous pain.
4. Group 4 (atypical MTrP group): participants had no active and no latent MTrP but at least one nodule on the low back muscle was visible in the ultrasound exam and had no spontaneous pain.

### Measurements

#### Demographic questionnaire

A structured interview was conducted to collect patient demographic information. Items such as date of birth, sex, ethnicity, race, marital status, and highest level of education completed were adopted from the National Healthy Worksite Demographic Questions Measure^16^. Participant’s employment status was adopted from the 2021 Behavioral Risk Factor Surveillance System Questionnaire^17^.

#### Pain pressure threshold (PPT)

PPT was used to objectively measure participant pain for each MTrP^18^. The Wagner Algometer FPX 25 (Wagner Instruments, Greenwich, CT) was utilized to apply and measure pressure. Patients were instructed to say “Stop” when they felt pain. This pain was assessed in pounds of force (lbf). PPT was measured three times at each MTrP. The three measurements were averaged and utilized for the PPT score for each MTrP.

#### Five repetition-sit to stand test (5R-STS)

(5R-STS) is a reliable^19^, clinical assessment used to objectively test movements used for everyday life (i.e., sitting down and standing up). Beginning in a sitting position, participants were instructed to stand up fully and sit down firmly on the seat five times as fast as possible on the command “go.” Researchers recorded the time using a stopwatch from the time “go” was announced to the time the participant sat down for the fifth time^19,20^.

#### Timed up and go (TUG)

The TUG assessment is a quick, objective measurement to assess physical function that demonstrates good test–retest reliability^21^. The assessment begins with the participant sitting in a chair. On the command “go,” the participant is to walk three meters at a regular pace, turn around, and walk back to the chair, returning to a sitting position. The staff recorded the time it took to complete this task using a stopwatch^21^.

#### Short form health outcomes survey (SF-20)

The SF-20 is a twenty-item measure that is made up of six health concepts including physical functioning (6 items), role functioning (2 items), social functioning (1 item), mental health (5 items), health perceptions (5 items), and pain (1item). Each response item is assigned a value of one to five. Scoring is achieved by changing the raw score. Reliability coefficients range from 0.81 to 0.88^22^, which are similar to the full-length versions. Cronbach alpha reliability for the current study ranged between 0.75 and 0.93, except the mental health (0.47).

#### Oswestry Disability Index (ODI)

The ODI evaluates disability due to lower back pain. It consists of ten items to assess pain intensity, personal care, lifting, walking, sitting, standing, sleeping, sex life, social life, and traveling^23^. Each response item is assigned a value of 0–5. The scoring is then conducted by adding up each item’s score and then dividing that total number by the total amount possible and multiplying by 100. A lower percentage represents a better low back health score whereas a higher percentage represents worse back health. The Cronbach’s Alpha reliability within adult populations were 0.76–0.88^24,25^. Cronbach alpha reliability for the current study was 0.74.

#### Pain Catastrophizing Scale (PCS)

The PCS assesses catastrophic thinking regarding pain^26^. This 13-item self-report scale includes three subscales: rumination, magnification and helplessness. The items are rated on a 5-point scale ranging from 0 (not at all) to 4 (all the time). Scoring consists of summing all the item scores to calculate a total score, which can range from 0 to 52. A higher score is associated with more pain catastrophizing. Cronbach’s alpha coefficients were 0.89–0.95^27,28^. Cronbach alpha reliability for the current study was 0.92.

#### Pain Self-Efficacy Questionnaire (PSEQ)

The PSEQ asks participants to rate how confident they are in performing various activities, despite their current pain. It is a 10-item measure, and responses are made on a 7-point scale ranging from 0 (not at all confident) to 6 (completely confident). The scale is scored by summing all the items to produce a total score ranging from 0 to 60, where higher scores indicate higher levels of self-efficacy beliefs. This measure has previously been found to be reliable with a Cronbach’s alpha coefficient of 0.94^29^. The Cronbach alpha reliability for the current study was 0.90.

#### Tampa Scale of Kinesiophobia (TSK)

The TSK assessed participant’s fear for movement and/or re-injury^30^. The measure includes 17-items using a 4-point Likert-type scale ranging from 1 (strongly disagree) to 4 (strongly agree). Four items are inversely scored. After these scores have been transformed, the item scores are added together so that a higher score suggests more fear. The reliability for this scale has been shown with a Cronbach’s alpha of 0.76^30^. The Cronbach alpha reliability for the current study was 0.76.

#### The Depression, Anxiety, and Stress Scale (DASS-21)

The DASS-21 is a self-reported measure to assess the emotional states of depression, anxiety, and stress^31^. It consists of 21 items with seven items belonging to each subscale (Stress, Anxiety, and Depression). A 4-point response scale is used ranging from 0 (did not apply to me at all) to 3 (applied to me very much or most of the time). Scoring is conducted by adding up each item belonging to the three different subscales and multiplying by 2 for each final score. In previous work, Cronbach’s alpha coefficients for each subscale were between 0.82 and 0.94^31,32^. Cronbach alpha reliability coefficients for the current study were 0.77, 0.70, and 0.45 for Stress, Anxiety, and Depression subscales, respectively.

#### Exercise Motivation Inventory-2 (EMI-2)

The EMI-2 is a 51-item, self-report measure designed to assess motives for exercising^33,34^. The measure includes a 5-point response scale where 0 indicates “Not at all true for me” and 5 indicates “Very true for me.” Scoring is achieved by adding the respective items and then finding the mean of these totals for each subscale. The inventory has 14 subscales, including Stress Management, Revitalization, Enjoyment, Challenge, Social Recognition, Affiliation, Competition, Health Pressures, Health Avoidance, Positive Health, Weight Management, Appearance, Strength & Endurance, and Nimbleness. Cronbach’s alpha coefficients for each subscale have been found to be between 0.66 and 0.95^33,35^. Cronbach alpha reliability coefficients for the current study were equal to or above 0.80 except for Social Recognition (0.78) and Health Pressures (0.64) subscales.

#### The body mass index

The body mass index (BMI) was determined by participants height and weight, using the formula: weight (lb)/[height (in)]^2^ × 703^36^.

#### Pain

Three items were adopted from the Brief Pain Inventory^37^ including patient reports of their current low back pain, best pain level (i.e., lowest pain rating) and the worst pain level (i.e., highest pain rating) they have had in the past 24 h. Using the Numeric Pain Rating Scale^38^, a visual scale ranging from 0 (no pain) to 10 (worst pain imaginable), participants were asked to rate each of these pain levels regarding the pain in their low back.

### Data management and analysis

After completing the data collection, the project manager entered the de-identified data into Excel worksheets. The project statistician evaluated if the data was entered correctly and made corrections if needed. The project statistician then imported the Excel worksheets into the Statistics Package for the Social Sciences for Windows version v28.0 (IBM Corp., Armonk, N.Y., USA) for data analysis.

A series of Chi-square Independence Tests were used to examine the distribution of each variable among different groups of participants, such as gender, ethnicity, educational level, etc. A series of one-way ANOVAs was conducted to examine the mean differences of each continuous demographic variable as well as the mean scores of subscales among the four groups of participants, such as age, BMI, the level of tenderness, etc. Finally, a series of Factorial ANCOVAs, using age, gender, and ethnicity (dummy coded) as the covariate, were conducted to examine if there were twitching or pain main effects, and the interaction effect on each scale.

## Results

### Descriptive results

Twenty-five participants with low back pain participated in this pilot study. Table 1 describes the demographic characteristics and general health information of the study sample and subgroups, with no significant differences between groups found. Overall, the mean number of MTrPs found on the low back was 4.36 (*SD* = 0.91) (Table 2). The mean reported tenderness level of MTrPs, when palpated, was 3.97 (*SD* = 1.43) on a numeric rating scale of 0–10, but the MTrP with the most severe tenderness level had a mean of 5.44 (*SD* = 1.47). The mean pressure pain threshold on MTrPs was 4.43 lbf (*SD* = 2.23) and the most sensitive MTrPs only tolerated 3.63 lbf (*SD* = 1.89).

**Table 1:** Demographic characteristics of the study sample (*N* = 25).

Variables	Total (*N* = 25)			Group 1 (*n* = 13)			Group 2 (*n* = 5)			Group 3 (*n* = 2)		Group 4 (*n* = 5)		*p* value
	*n*	%		*n*	%		*n*	%		*n*	%	*n*	%	
Sex														χ^2^(3) = 3.79, *p* = .29
Male	7	28.0		5	38.5		0	0.0		0	0.0	2	40.0	
Female	18	72.0		8	61.5		5	100.0		2	100.0	3	60.0	
Ethnicity														χ^2^(6) = 2.91, *p* = .82
White	16	64.0		9	69.2		3	60.0		1	50.0	3	60.0	
Black/African American	3	12.0		2	15.4		1	20.0		0	0.0	0	0.0	
Asian	6	24.0		2	15.4		1	20.0		1	50.0	2	40.0	
Employment														χ^2^(3) = 1.76, *p* = .62
Employed	15	60.0		8	61.5		4	80.0		1	50.0	2	40.0	
Unemployed/students	10	40.0		5	38.5		1	20.0		1	50.0	3	60.0	
Education														χ^2^(3) = 0.96, *p* = .81
Some college	1	4.0		1	7.7		0	0.0		0	0.0	0	0.0	
College graduate	24	96.0		12	92.3		5	100.0		2	100.0	5	100.0	
Marital status														χ^2^(6) = 4.42, *p* = .62
Married	18	72.0		10	76.9		3	60.0		2	100.0	3	60.0	
Unmarried couple	3	12.0		2	15.4		1	20.0		0	0.0	0	0.0	
Never married	4	16.0		1	7.7		1	20.0		0	0.0	2	40.0	
Health														χ^2^(9) = 9.74, *p* = .37
Excellent	3	12.0		2	15.4		0	0.0		0	0.0	1	20.0	
Very good	9	36.0		5	38.5		2	40.0		0	0.0	2	40.0	
Good	11	44.0		6	46.2		3	60.0		1	50.0	1	20.0	
Fair	2	8.0		0	0.0		0	0.0		1	50.0	1	20.0	
Pain in past 4 weeks														χ^2^(6) = 8.99, *p* = .17
Very mild	7	28.0		2	15.4		1	20.0		0	0.0	4	80.0	
Mild	10	40.0		6	46.2		2	40.0		1	50.0	1	20.0	
Moderate	8	32.0		5	38.5		2	40.0		1	50.0	0	0.0	
Pain medication for MPS														χ^2^(3) = 6.73, *p* = .08
Yes	20	80.0		12	92.3		4	80.0		2	100.0	2	40.0	
No	5	20.0		1	7.7		1	20.0		0	0.0	3	60.0	
Mean age (SD)	34.56 (10.75)			34.0 (11.71)			34.80 (7.29)			34.50 (17.68)		35.60 (12.18)		*F*(3,21) = 0.02*, p* = 1.00
Mean BMI (SD)	26.84 (5.45)			28.68 (6.32)			24.45 (3.31)			27.59 (3.66)		24.17 (4.12)		*F*(3,21) = 1.26*, p* = .31
Mean numbers of current health conditions (SD)	1.52 (1.29)			1.42 (1.38)			1.67 (2.08)			2.50 (0.71)		1.25 (0.50)		*F*(3,21) = 0.44*, p* = .73
Average pain duration in month (SD)	82.8 (85.73)			60.46 (75.74)			120.00 (83.57)			36.00 (33.94)		122.4 (114.91)		*F*(3,21) = 1.19*, p* = .34

**Table 2:** Results of clinical assessment and questionnaire intakes by groups (*N* = 25).

Scale	Total		Group 1 (*n* = 13)		Group 2 (*n* = 5)		Group 3 (*n* = 2)		Group 4 (*n* = 5)		*F* (3,21)	*p*
	*M*	*SD*	*M*	*SD*	*M*	*SD*	*M*	*SD*	*M*	*SD*		
MTrPs												
Number	4.36	0.91	4.62	0.77	4.00	0.71	5.50	0.71	3.60	0.89	3.83	***.***03*
Duration (in month)	82.80	85.73	60.46	75.74	120.00	83.57	36.00	33.94	122.40	114.91	1.19	.34
Tenderness (average)	3.97	1.43	4.30	1.61	3.46	0.84	5.00	1.88	3.20	0.96	1.33	.29
Tenderness (severe)	5.44	1.47	5.69	1.49	5.00	1.58	6.50	2.12	4.80	1.10	0.93	.45
PPT (average)	4.43	2.23	4.12	2.61	4.21	2.05	3.50	0.90	5.79	1.33	0.82	.50
PPT (severe)	3.63	1.89	3.33	2.29	3.42	1.34	3.20	0.71	4.79	1.29	0.77	.53
STS	10.60	2.48	10.30	2.46	11.86	3.27	10.99	0.78	9.96	2.25	0.59	.63
TUG	9.36	1.76	9.49	1.90	9.32	1.16	10.96	1.86	8.43	1.80	1.04	.40
SF-20 (%)												
Physical function	83.34	18.94	85.26	16.37	73.34	27.25	62.50	5.94	96.68	4.55	2.56	.08
Role function	88.00	25.12	88.46	29.96	85.00	22.36	75.00	35.36	95.00	11.18	0.30	.82
Social function	92.80	11.37	92.31	13.01	88.00	10.95	90.00	14.14	100.00	0.00	1.02	.41
Mental health	78.48	9.98	76.62	11.18	78.02	9.12	82.00	2.83	82.40	10.04	0.46	.71
Health perceptions	80.28	12.37	81.47	11.16	75.96	15.38	66.60	5.09	86.16	12.71	1.25	.32
Pain	40.80	15.79	44.62	14.50	44.0	16.73	50.00	14.14	26.00	8.94	3.06	.05
ODI	11.44	7.80	14.00	8.12	13.20	6.72	10.00	2.83	3.60	4.10	2.75	.07
PCS	11.48	9.75	8.15	5.21	15.60	9.63	8.00	8.49	17.40	16.46	1.62	.22
PSEQ	53.52	6.63	53.85	5.16	48.40	10.41	58.00	1.41	56.00	4.95	1.67	.20
TSK	32.60	6.34	31.15	5.97	36.80	8.70	36.00	1.41	30.80	4.38	1.34	.29
DASS 21												
Stress	7.92	6.84	9.38	5.38	4.00	4.00	4.00	5.66	9.60	11.52	1.07	.38
Anxiety	3.44	5.43	3.23	4.87	1.20	1.79	6.00	8.49	5.20	8.44	0.58	.63
Depression	1.76	2.11	1.54	1.45	0.40	0.89	3.00	4.24	3.20	3.03	1.96	.15
EMI												
Stress management	3.13	1.63	3.02	1.56	3.40	1.57	3.63	0.53	2.95	2.40	0.13	.94
Revitalization	2.88	1.62	2.79	1.56	3.00	1.72	3.17	0.24	2.87	2.34	0.04	.99
Enjoyment	2.51	1.86	2.35	1.74	2.90	2.19	2.25	1.06	2.65	2.52	0.11	.95
Challenge	2.08	1.50	2.27	1.62	1.80	1.24	2.00	1.06	1.90	1.88	0.14	.94
Social recognition	1.00	0.93	1.06	1.05	1.25	0.95	0.50	0.71	0.80	0.78	0.37	.77
Affiliation	1.49	1.29	1.02	0.96	2.05	1.57	2.13	1.59	1.90	1.60	1.27	.31
Competition	1.58	1.61	1.56	1.52	1.65	1.90	1.00	0.71	1.80	2.15	0.11	.95
Health pressures	2.03	1.43	1.90	1.33	2.20	1.28	2.33	0.94	2.07	2.23	0.08	.97
Health avoidance	3.83	1.08	3.79	0.84	3.87	1.12	4.33	0.47	3.67	1.84	0.17	.92
Positive health	4.24	0.94	4.31	0.60	4.27	0.98	4.83	0.24	3.80	1.66	0.63	.60
Weight management	3.99	1.32	3.56	1.64	4.20	0.96	4.50	0.00	4.70	0.33	1.10	.37
Appearance	3.12	1.22	2.69	1.42	3.25	0.68	3.38	0.18	4.00	0.85	1.56	.23
Strength & endurance	3.66	1.33	3.94	0.79	3.65	1.42	3.13	1.59	3.15	2.32	0.51	.68
Nimbleness	3.15	1.31	3.23	1.05	3.33	0.53	3.17	0.24	2.73	2.52	0.20	.90
Numeric pain rating												
Current pain	2.96	1.95	3.69	1.89	2.80	1.10	4.50	0.71	0.60	0.89	5.42	.01*
Best pain past 24 h	1.04	1.40	1.23	1.59	0.80	1.30	2.00	1.41	0.40	0.89	0.77	.52
Worst pain past 24 h	5.00	2.57	6.23	1.83	4.60	2.70	6.50	0.71	1.60	1.14	7.71	.001**

*PPT* pain pressure threshold, *STS* five repetition sit-to-stand test, *TUG* timed up and go, *ODI* Oswestry Disability Index, *PCS* Pain Catastrophizing Scale, *PSEQ* Pain Self-Efficacy Questionnaire, *TSK* Tampa Scale of Kinesiophobia. *p < 0.05; **p < 0.01; ***p < 0.001.

Objective measure of physical function, STS, was 10.60 (*SD* = 2.48) seconds and the mean of TUG was 9.36 (*SD* = 1.76) seconds. The physical health function, role functioning, social functioning, mental health, health perceptions, and the pain component of quality-of-life measure were 83.34% (*SD* = 18.94%), 88.00% (*SD* = 25.12%), 92.80% (*SD* = 11.37%*),* 78.48% (*SD* = 9.98%), 80.28% (*SD* = 12.37%), and 40.80% (*SD* = 15.79%), respectively. The self-reported disability measure, ODI, was 11.44 (*SD* = 7.80). The PCS score for the study sample suggests participants experience moderate pain catastrophizing (*M* = 11.48, *SD* = 9.75). The participant’s average pain self-efficacy beliefs score, PSEQ, was 53.52 (*SD* = 6.63) (i.e., they are much more confident in their ability to handle pain)*.* The mean score on the TSK for the study’s sample is 32.60 (*SD* = 6.34), suggesting the study sample is less fearful of movement or reinjury. Stress, anxiety and depression scores of the emotional health measure (DASS 21) were 7.92 (*SD* = 6.84), 3.44 (*SD* = 5.43) and 1.76 (*SD* = 2.11), respectively, meaning on average participants reported a normal level of stress, anxiety, and depression^39^. The mean exercise motivation, EMI subscales were between 1.00 and 4.24 with a standard deviation between 0.93 and 1.86. The levels of current pain, best pain in the past 24 h, and worst pain within 24 h were 2.96 (*SD* = 1.95), 1.04 (*SD* = 1.40), and 5.00 (*SD* = 2.57) respectively, indicating that participants’ current and best pain within 24 h were categorized as “mild” while their worst reported pain was considered to be in the “moderate” cutoff^38^.

### Difference between patients in 4 groups

Results indicate no statistically significant differences in the four groups in clinical assessment and questionnaire intakes, except for the number of MTrPs and verbal report of pain (Table 2). Participants in spontaneous pain without twitching (Group 3), had more trigger points than those in the group who had no spontaneous pain and no twitching (Group 4), *F*(3.21) = 3.83, *p* = 0.03. In addition, participants from different groups reported different levels of current pain and worst pain in the past 24 h, *F*(3,21) = 5.42, *p* = 0.01; *F*(3,21) = 7.71, *p* = 0.001, respectively. The Bonferroni post hoc test indicated that participants with pain [Group 1 (active MTrP) and Group 3 (atypical MTrP)] reported significantly higher levels of current pain compared with the participants in Group 4, *p* = 0.04, *p* = 0.01, respectively. Similarly, participants in Group 1 and Group 3 also reported higher levels of worst pain in the past 24 h compared with participants in Group 4, *p* < 0.001, *p* = 0.03, respectively.

### Pain report, twitching response, and interaction on patients reported outcomes

**Figure 1:**
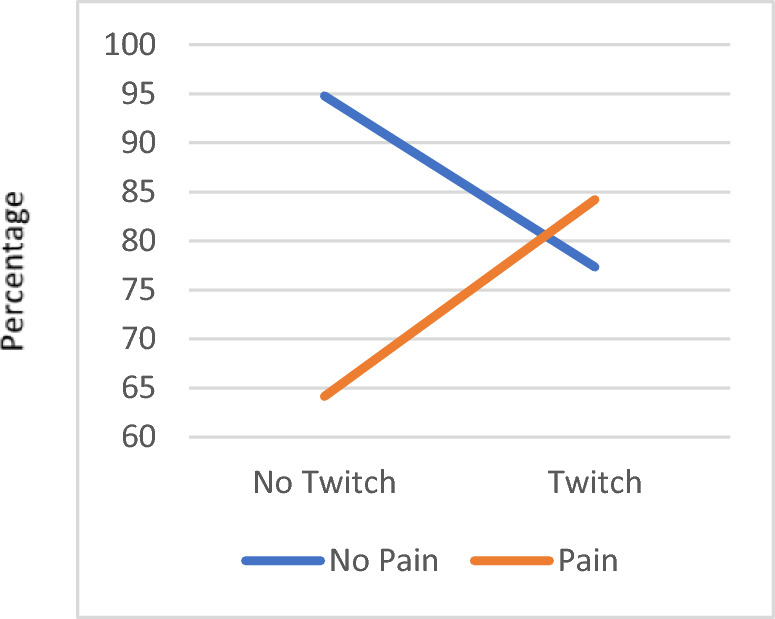
The interaction effect in the SF-20 physical function subscale between pain and twitching. For participants who reported pain, their SF-20 Physical Function response was lower if they did not have twitching responses (64.15 ± 11.35), while it was higher if they had twitching response (84.20 ± 4.41). On the other hand, for those who did not report pain, their response in SF-20 Physical Function was higher if they did not have twitching responses (94.78 ± 7.27) and lower if they had twitching responses (77.34 ± 7.40). There was a significant difference in the SF-20 Physical Function percentage between Pain and No Pain participants when they had no twitching responses, *p* = .04.

**Figure 2:**
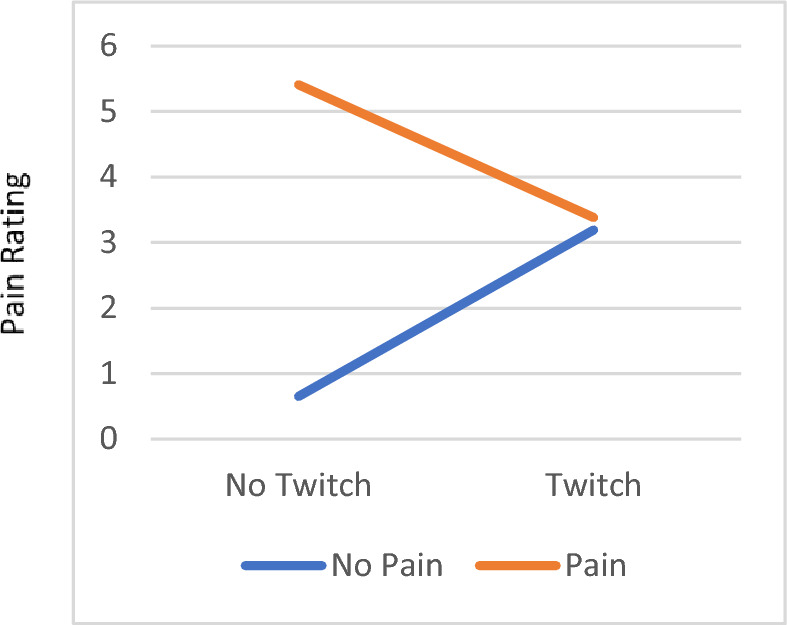
The interaction effect in the reported current pain level on the numeric pain rating scale between pain and twitching. For participants who reported pain, their reported Current Pain Level was higher if they did not have twitching responses (5.41 ± 0.87), while it was lower if they had twitching response (0.38 ± 0.34). However, for those who did not report pain, their Current Pain Level was lower if they did not have twitching responses (0.65 ± 0.54) and higher if they had twitching responses (3.19 ± 0.56). There was a significant difference in the Current Pain Level between pain and no pain participants when they had no twitching responses, *p* < .001.

**Figure 3:**
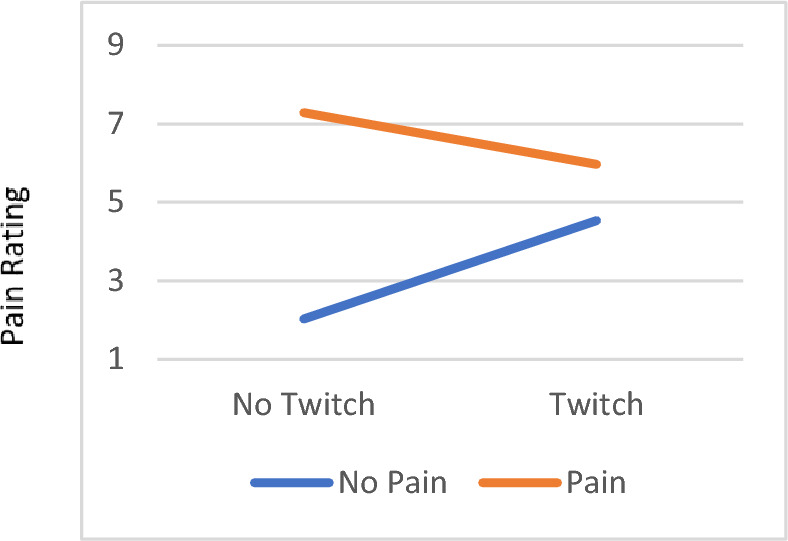
The interaction effect in the reported worst pain level in the past 24 h on the numeric pain rating scale between pain and twitching. For participants who reported pain, their Worst Pain Level in the past 24 h was higher if they did not have twitching responses (7.29 ± 1.19), while it was lower if they had twitching responses (5.97 ± 0.46). Conversely, for those who did not report pain, their Worst Pain Level in the past 24 h was lower if they did not have twitching responses (2.03 ± 0.77) and higher if they had twitching responses (4.54 ± 0.78). There was a significant difference in the current pain level between pain and no pain participants when they had no twitching responses, *p* = .04.

After controlling the covariates including age, gender, and race, results indicated there were interaction effects between pain report and twitching response on participants' reports of physical function, current pain level, and worst pain level in the past 24 h, *F*(1,17) = 5.10, *p* = 0.04; *F*(1,17) = 13.03, *p* = 0.002; *F*(1,17) = 4.79, *p* = 0.04, respectively (Tables 3 and 4). Participants who experienced spontaneous pain (Orange line), but no twitching (Group 3) reported lower levels of physical function (Fig. 1), higher levels of current pain (Fig. 2), and higher levels of worst pain (Fig. 3) in the past 24 h compared to those who had both spontaneous pain and twitching response (Group 1). However, participants who did not have spontaneous pain reported an opposite pattern (Blue line). They reported higher levels of physical function, lower levels of current pain, and lower levels of worst pain in the past 24 h if they had no twitching (Group 4) response compared to those who had a twitching response (Group 2).

**Table 3:** Clinical assessment and questionnaire intakes by twitching, pain, sex, and ethnicity groups.

Scale	Twitching				Spontaneous Pain				Sex				Ethnicity			
	Yes (*n* = 18)		No (*n* = 7)		Yes (*n* = 15)		No (*n* = 10)		Female (*n* = 18)		Male (*n* = 7)		Black (*n* = 3)		Others (*n* = 22)	
	*M*	*SD*	*M*	*SD*	*M*	*SD*	*M*	*SD*	*M*	*SD*	*M*	*SD*	*M*	*SD*	*M*	*SD*
MTrPs																
Number	4.44	0.78	4.14	1.21	4.73	0.80	3.80	0.79	4.33	0.91	4.43	0.98	4.67	0.58	4.32	0.95
Duration (in Month)	77.00	80.29	97.71	103.79	57.20	71.23	121.2	94.73	109.83	87.16	13.29	6.45	84.00	60.00	82.64	89.76
Tenderness (average)	4.07	1.47	3.71	1.41	4.40	1.60	3.33	0.86	3.65	0.98	4.81	2.08	5.00	2.73	3.83	1.21
Tenderness (severe)	5.50	1.50	5.29	1.50	5.80	1.52	4.90	1.29	5.22	1.31	6.00	1.83	6.67	1.53	5.27	1.42
PPT (average)	4.15	2.41	5.14	1.61	4.04	2.44	5.00	1.83	4.64	2.22	3.88	2.34	4.24	3.83	4.45	2.07
PPT (severe)	3.36	2.03	4.34	1.34	3.31	2.13	4.11	1.44	3.86	1.81	3.05	2.13	3.46	2.83	3.65	1.82
STS	10.74	2.70	10.26	1.93	10.39	2.30	10.91	2.83	10.32	2.40	11.32	2.72	14.02	1.40	10.14	2.22
TUG	9.44	1.69	9.15	2.06	9.68	1.90	8.88	1.50	9.50	1.75	9.00	1.88	11.58	1.17	9.06	1.62
SF-20																
Physical function	81.95	19.85	86.91	17.26	82.23	17.21	85.01	22.15	77.78	19.59	97.63	4.05	72.23	25.46	84.85	18.12
Role function	87.50	27.45	89.29	19.67	86.67	29.68	90.00	17.48	84.72	28.62	96.43	9.45	66.67	28.87	90.91	23.84
Social function	91.11	12.31	97.14	7.56	92.00	12.65	94.00	9.66	91.11	12.31	97.14	7.56	86.67	11.55	93.64	11.36
Mental health	77.01	10.40	82.29	8.28	77.33	10.55	80.21	9.33	76.12	9.70	84.57	8.46	72.70	10.97	79.27	9.85
Health perceptions	79.94	12.25	81.14	13.62	79.75	11.37	81.06	14.35	75.53	9.66	92.49	10.28	72.03	9.85	81.40	12.43
Pain	44.44	14.64	31.43	15.74	45.33	14.07	34.00	16.47	41.11	14.51	40.00	20.00	60.00	0.00	38.18	15.00
ODI	13.78	7.57	5.43	4.72	13.47	7.69	8.40	7.29	12.11	6.70	9.71	10.55	26.67	5.03	9.36	5.43
PCS	10.22	7.26	14.71	14.61	8.13	5.33	16.50	12.75	14.72	9.44	3.14	3.89	12.67	4.73	11.32	10.31
PSEQ	52.33	7.11	56.57	4.20	54.40	5.01	52.20	8.66	52.39	7.09	56.43	4.47	49.67	4.73	54.05	6.76
TSK	32.72	7.05	32.29	4.42	31.80	5.80	33.80	7.22	34.17	6.01	28.57	5.65	39.00	3.61	31.73	6.17
DASS 21																
Stress	7.89	5.51	8.00	10.07	8.67	5.54	6.80	8.65	8.44	7.96	6.57	2.23	9.33	1.16	7.73	7.29
Anxiety	2.67	4.28	5.43	7.72	3.60	5.14	3.20	6.12	4.22	6.13	1.43	2.23	2.00	3.46	3.64	5.68
Depression	1.22	1.40	3.14	3.02	1.73	1.83	1.80	2.57	2.11	2.32	0.86	1.07	0.67	1.16	1.91	2.18
EMI																
Stress management	3.13	1.52	3.14	2.00	3.10	1.47	3.18	1.93	2.89	1.48	3.75	1.95	1.83	0.14	3.31	1.66
Revitalization	2.85	1.56	2.95	1.92	2.84	1.45	2.93	1.94	2.63	1.52	3.52	1.81	1.11	1.39	3.12	1.52
Enjoyment	2.50	1.82	2.54	2.11	2.33	1.63	2.78	2.23	2.19	1.78	3.32	1.95	0.75	0.75	2.75	1.85
Challenge	2.14	1.50	1.93	1.59	2.23	1.53	1.85	1.50	1.88	1.40	2.61	1.73	0.50	0.50	2.30	1.46
Social recognition	1.11	1.00	0.71	0.71	0.98	1.01	1.03	0.85	0.90	0.89	1.25	1.07	0.08	0.14	1.13	0.93
Affiliation	1.31	1.21	1.96	1.46	1.17	1.06	1.98	1.49	1.32	1.26	1.93	1.35	0.50	0.43	1.63	1.31
Competition	1.58	1.58	1.57	1.82	1.48	1.44	1.73	1.91	1.18	1.47	2.61	1.59	0.33	0.58	1.75	1.64
Health pressure	1.98	1.29	2.14	1.86	1.96	1.27	2.13	1.72	1.87	1.50	2.43	1.24	3.44	0.69	1.83	1.40
Health avoidance	3.81	0.89	3.86	1.55	3.87	0.81	3.77	1.44	3.69	1.20	4.19	0.63	4.00	0.88	3.80	1.12
Positive health	4.30	0.69	4.10	1.45	4.38	0.59	4.03	1.31	4.07	1.01	4.67	0.58	4.22	0.69	4.24	0.98
Weight management	3.74	1.48	4.64	0.28	3.68	1.55	4.45	0.72	4.10	1.30	3.71	1.45	3.33	1.44	4.08	1.31
Appearance	2.85	1.27	3.82	0.76	2.78	1.34	3.63	0.83	3.38	0.95	2.46	1.64	2.00	1.80	3.27	1.09
Strength & endurance	3.86	0.97	3.14	2.00	3.83	0.89	3.40	1.83	3.42	1.43	4.29	0.81	3.92	1.04	3.63	1.38
Nimbleness	3.26	0.92	2.86	2.07	3.22	0.97	3.03	1.75	3.07	1.32	3.33	1.36	3.11	1.02	3.15	1.36
Numeric pain rating																
Current pain	3.44	1.72	1.71	2.06	3.80	1.78	1.70	1.49	2.50	1.42	4.14	2.67	4.67	2.08	2.73	1.86
Best pain past 24 h	1.11	1.49	0.86	1.21	1.33	1.54	0.60	1.07	1.00	1.24	1.14	1.86	4.00	1.00	0.64	0.85
Worst pain past 24 h	5.78	2.16	3.00	2.58	6.27	1.71	3.10	2.51	4.72	2.35	5.71	3.15	7.67	1.53	4.64	2.48

*PPT* pain pressure threshold, *STS* five repetition sit-to-stand test, *TUG* timed up and go, *ODI* Oswestry Disability Index, *PCS* Pain Catastrophizing Scale, *PSEQ* Pain Self-Efficacy Questionnaire, *TSK* Tampa Scale of Kinesiophobia.

**Table 4:** Factorial ANCOVA results (*N* = 25, *df* = 1,17).

Scale	Covariates						Factors					
	Age		Sex		Black		Twitching		Spontaneous pain		Twitch × pain	
	*F*	*p*	*F*	*p*	*F*	*p*	*F*	*p*	*F*	*p*	*F*	*p*
MTrPs												
Number	1.06	.32	1.23	.28	0.11	.75	0.14	.72	***11.11***	.004**	3.66	.07
Duration (in month)	1.05	.32	***4.48***	.05*	0.09	.77	0.87	.37	***5.93***	.03*	1.94	.18
Tenderness (average)	0.09	.76	3.23	.09	1.54	.23	0.28	.61	4.08	.06	1.70	.21
Tenderness (severe)	0.04	.85	1.29	.27	2.00	.18	0.40	.54	2.76	.12	1.00	.33
PPT(average)	0.49	.50	1.73	.21	0.09	.76	0.58	.46	0.97	.34	1.60	.22
PPT severe	1.19	.29	2.09	.17	0.19	.67	1.04	.32	0.65	.43	1.27	.28
STS	0.42	.53	1.11	.31	***6.48***	.02*	0.03	.86	0.03	.86	2.07	.17
TUG	3.58	.07	0.01	.93	3.20	.09	0.83	.38	3.90	.07	2.09	.17
SF-20												
Physical function	3.02	.10	2.04	.17	0.25	.62	0.03	.87	2.39	.14	***5.10***	.04*
Rold function	4.35	.02	0.01	.92	1.34	.26	0.01	.92	0.79	.39	0.96	.34
Social function	1.35	.26	0.41	.53	0.03	.87	0.28	.61	0.32	.58	0.92	.35
Mental health	0.59	.46	4.31	.05	1.58	.23	0.69	.42	0.02	.88	0.99	.33
Health perceptions	0.003	.96	***7.30***	.02*	3.32	.09	0.06	.81	1.67	.21	1.18	.29
Pain	1.68	.21	< .001	1.00	***6.43***	.02*	0.43	.52	4.10	.06	2.69	.12
ODI	1.58	.23	0.79	.39	***19.35***	< .001***	2.88	.11	3.15	.09	0.47	.50
PCS	0.09	.77	***6.76***	.02*	1.14	.30	0.01	.92	***5.55***	.03*	2.07	.17
PSEQ	0.01	.91	0.71	.41	0.57	.46	2.51	.13	1.23	.28	0.01	.92
TSK	0.10	.76	1.17	.30	3.54	.08	0.001	.98	0.001	.97	1.54	.23
DASS 21												
Stress	0.09	.77	1.76	.20	0.13	.72	0.05	.83	< 0.001	1.00	3.75	.07
Anxiety	0.27	.61	2.49	.13	0.17	.68	1.95	.18	0.29	.60	0.48	.50
Depression	1.33	.27	2.38	.14	0.52	.48	4.04	.06	0.27	.61	1.44	.25
EMI												
Stress management	0.59	.45	2.47	.13	3.17	.09	0.04	.85	0.04	.86	1.70	.21
Revitalization	0.18	.67	1.54	.23	***5.36***	.03*	0.01	.94	0.003	.96	0.90	.36
Enjoyment	0.01	.94	1.45	.24	3.78	.07	0.10	.76	0.31	.58	0.48	.50
Challenge	2.14	.16	0.26	.62	2.58	.13	0.15	.70	0.09	.77	< .001	1.00
Social recognition	1.64	.22	0.84	.37	2.35	.14	2.70	.12	0.47	.50	0.05	.83
Affiliation	0.23	.64	3.52	.08	2.32	.15	0.18	.67	0.46	.51	3.09	.10
Competition	3.37	.08	3.86	.07	0.76	.40	0.57	.46	0.56	.46	0.05	.82
Health pressures	2.79	.11	2.30	.15	1.35	.26	0.23	.64	0.01	.92	0.93	.35
Health avoidance	1.50	.24	1.54	.23	0.12	.74	0.18	.68	0.36	.56	1.35	.26
Positive health	0.25	.62	2.60	.13	0.23	.64	0.01	.94	1.33	.26	2.68	.12
Weight management	1.07	.32	0.71	.41	0.05	.82	1.16	.30	0.46	.51	< .001	1.00
Appearance	0.62	.44	4.18	.06	1.08	.31	1.30	.27	1.34	.26	0.62	.44
Strength & endurance	0.02	.89	1.24	.28	0.001	.98	0.67	.43	0.04	.85	0.06	.82
Nimbleness	0.08	.78	0.13	.73	0.16	.70	0.10	.75	0.06	.81	0.29	.60
Numeric pain rating												
Current pain	0.21	.65	***8.92***	***.01***	1.54	.23	0.17	.68	***17.83***	< .001***	***13.03***	.002**
Best pain past 24 h	1.34	.26	0.00	.98	***33.05***	< .001***	***4.98***	***.04***	***8.45***	.01*	1.79	.20
Worst pain past 24 h	0.14	.72	0.46	.51	3.56	.08	0.48	.50	***17.10***	< .001***	***4.79***	.04*

*PPT* pain pressure threshold, *STS* five repetition sit-to-stand test, *TUG* timed up and go, *ODI* Oswestry Disability Index, *PCS* Pain Catastrophizing Scale, *PSEQ* Pain Self-Efficacy Questionnaire, *TSK* Tampa Scale of Kinesiophobia.

Significant values are in bold. *p < 0.05; **p < 0.01; ***p < 0.001.

Participants with twitching responses reported a higher level of best pain score in the past 24 h compared to those who did not have twitching responses (*F*[1, 17] = 4.98, *p* = 0.04; *M* = 1.11 ± 1.49 vs. *M* = 0.86 ± 1.21). This indicates that participants in Group 1 (active) and Group 2 (latent) report higher levels of best pain in the past 24 h as compared to those with atypical MTrPs (Group 3 and Group 4). Further, participants in groups which reported spontaneous pain (Groups 1 and 3) also had more MTrPs (*F*[1, 17] = 11.11, *p* = 0.004; *M* = 4.73 ± 0.80 vs. *M* = 3.80 ± 0.79); shorter period of pain history (*F*[1, 17] = 5.93, *p* = 0.03; *M* = 57.20 months ± 71.23 months vs. *M* = 121.20 months ± 94.73 months); low PCS total scores (*F*[1, 17] = 5.55, *p* = 0.03; *M* = 8.13 ± 5.33 vs. *M* = 16.50 ± 12.75); higher level of current pain (*F*[1, 17] = 17.83, *p* < 0.001; *M* = 3.80 ± 1.78 vs. *M* = 1.70 ± 1.49); best pain level in the past 24 h (*F*[1, 17] = 8.45, *p* = 0.01; *M* = 1.33 ± 1.54 vs. *M* = 0.60 ± 1.07); and worst pain level in the past 24 h (*F*[1, 17] = 17.10, *p* < 0.001; *M* = 6.27 ± 1.71 vs. *M* = 3.10 ± 2.51) compared to those who were in groups that did not report being in spontaneous pain (Groups 2 and 4).

Female participants had a longer history of pain (*F*[1, 17] = 4.48, *p* = 0.05; *M* = 109.83 months ± 87.16 months vs. *M* = 13.29 months ± 6.45 months), lower level of health perceptions (*F*[1, 17] = 7.30, *p* = 0.02; *M* = 75.53 ± 9.66 vs. *M* = 92.49 ± 10.28), higher PCS score (*F*[1,17] = 6.76, *p* = 0.02; *M* = 14.72 ± 9.44 vs. *M* = 3.14 ± 3.89), and lower level of current pain (*F*[1.17] = 8.92, *p* = 0.01; *M* = 2.50 ± 1.42 vs. *M* = 4.14 ± 2.67) than male participants (Tables 3 and 4). In addition, Black participants had worse performance in STS (*F*[1, 17] = 6.48, *p* = 0.02; *M* = 14.02 ± 1.40 vs. *M* = 10.14 ± 2.22), a higher pain component in the quality of life measure (*F*[1, 17] = 6.43, *p* = 0.02; *M* = 60.00 ± 0.00 vs. *M* = 38.18 ± 15.00), higher ODI scores (*F*[1, 17] = 19.35, *p* < 0.001; *M* = 26.67 ± 5.03 vs. *M* = 9.36 ± 5.43), lower EMI revitalization scores (*F*[1, 17] = 5.36, *p* = 0.03; *M* = 1.11 ± 1.39 vs. *M* = 3.12 ± 1.52), and higher levels of best pain in the past 24 h (*F*[1, 17] = 33.05, *p* < 0.001; *M* = 4.00 ± 1.00 vs. *M* = 0.64 ± 0.85) compared with other races. The age of the participants had no impact on each measure.

## Discussion

This is one of few studies investigating characteristics of patients with MPS of the low back. This study identified several important findings that could potentially assist in understanding MPS of the low back. A similar study with a non-US population revealed 63.5–90% of patients with low back pain have MPS^11,12^. The current study found that 100% of the low back pain patients studied had more than one MTrP visible using ultrasound on the low back muscle.

Overall, participants in the study were not afraid of movement, reported a normal level of mental distress, and experienced mild to moderate pain. The participants reported an overall better quality of life^40^, less fearful to move^41^, greater pain self-efficacy^42^ and better disability score^23^ when compared with a sample of similarly aged participants who have been diagnosed with low back pain. Our participants had moderate pain catastrophizing scores, similar to populations with chronic low back pain in prior studies^26,43^. They had similar levels of motivation for exercising^44^, but it took the study sample longer to complete the physical function measures (such as STS^45^ and TUG^46^) as compared to a healthy population without low back pain.

The current study found that not all MTrPs or nodules identified met the definitions provided by Travell and Simons^2^. There are a couple of potential explanations: first, several past studies have pointed out that manual palpation for identifying MTrPs is not as reliable as what we would expect for a diagnostic option^47,48^. One recent study shows the inter-rater reliability between 0.49 and 0.75 for low leg muscles^48^. In addition, a systematic review study found the MTrPs diagnostic criteria utilized for clinical trials were widely different. A 2020 review suggested the most popular combination is spot tenderness, referred pain, and local twitch response^49^. Potentially, patients with atypical MTrP symptoms could be misdiagnosed due to improper palpation methods or variation in definitions.

It is possible that atypical MTrPs may be in the process of becoming active or latent MTrPs, or dissolving as the disease process is ending in that area and returning to normal. Alternatively, one study postulated atypical MTrPs are also a sign of MPS and proposed additional diagnostic criteria^50^. This was also demonstrated in the current study. Our results revealed trends that Group 3 (atypical, spontaneous pain and no twitching group) had the highest total number of MTrPs, level of current pain, and highest level of worse pain within 24 h; followed by Group 1 (active), Group 2 (latent) and Group 4 (atypical, no spontaneous pain and no twitching). However, the only significant difference in number of MTrPs was between Group 3 and Group 4. In addition, Group 3 also showed a significantly lower level of physical function compared to Group 4. That is, participants with spontaneous pain but no twitching (Group 3) had more MTrPs, worse pain within 24 h, and poor self-reported physical function compared to those who reported no spontaneous pain and no twitching (Group 4). Additional studies are needed, including finding objective ways to identify MTrPs and define MPS.

Participants in this study exhibited multiple MTrPs and mixed numbers of MTrPs similar to other studies examining patients with low back pain, as well as studies of patients with fibromyalgia^14,51^. One study of non-specific low back pain patients and a matching control group found patients with non-specific low back pain had an average of 5.5 (*SD* = 1.9) mixed MTrPs, where 3.5 (*SD* = 2.3) were active and 2 (*SD* = 1.5) were latent. Comparatively, the matched control group had an average of 1 (*SD* = 1.5) latent MTrPs, and no active MTrPs^14^. One study also found that women with fibromyalgia had an average of 11 MTrPs, with the majority of them active MTrPs, while healthy control patients only had latent MTrPs with an average of two MTrPs^51^.

Participants with spontaneous pain, with or without twitching reports had the tendency to have more MTrPs compared to those who reported no spontaneous pain, regardless of having twitching response. An earlier study with low back pain patients also found the number of active MTrPs was associated with pain intensity^14^. In a patient population with fibromyalgia, multiple MTrPs seem to relate to having a pain complaint^51^. This suggests the number of MTrPs may be an important predictor for the level of pain complaints in MPS patients. In addition, these studies show that MPS probably is not a unique symptom with a specific musculoskeletal disorder but a common component of several musculoskeletal pain conditions.

Pain complaint seems to be a decisive factor associated with lower physical function, but twitching response is not. This study found participants who reported spontaneous pain regardless of the twitching status had a shorter duration of MPS, higher pain catastrophizing scores, and higher pain report levels compared to those who reported no spontaneous pain with or without twitching status. Rationale for these results include the potential role of pain catastrophizing on these outcomes since past studies have revealed a higher level of pain catastrophizing was associated with developing higher pain intensity and higher levels of disability in patients with temporomandibular dysfunction, a condition commonly caused by MPS^52^. However, the causal relationship between pain reports and catastrophizing in this crossover study cannot be determined. Alternatively, although MPS is chronic in nature, spontaneous pain may indicate participants are in a more acute stage of the pain spectrum than those who have no spontaneous pain leading to higher pain catastrophizing scores and pain report.

We found that sex and ethnicity may be important covariates that affect the manifestation of MPS. Female participants tended to have a longer history of pain, higher catastrophizing scores, and lower levels of current pain than male participants. This study only included a few Black participants. However, we found that black participants had worse physical function, higher level of disability, and higher level of best pain in the past 24 h compared with other races. No studies were found for an MPS population, but in patients with low back pain, similar findings were reported in which Black participants with chronic low back pain also reported a higher level of pain severity^53,54^ and higher levels of disability compared to White participants, but the study did not find a gender difference in pain reporting^54,55^.

Limitations of this study include a small sample size; thus, findings should be considered cautiously. In addition, the cross-sectional design of this study limits its ability to make causal inferences. Finally, this is a highly educated group of participants, therefore the results cannot be generalized to other populations.

These findings suggest clinicians should examine patients with low back pain for multiple MTrPs and consider that a mixture of different types of MTrPs may be contributing to the condition. A wide definition of MTrP should be used, with a focus on spontaneous pain and decreased physical function, rather than a twitch response. Finally, palpation skills training is important for assessing MTrPs until more reliable methods can be developed.

## Data Availability

The datasets generated during and/or analyzed during the current study are available from the corresponding author on reasonable request.
